# Cost and Relative Value of Road Kill Surveys for Enhanced Rabies Surveillance in Raccoon Rabies Management

**DOI:** 10.3390/tropicalmed2020013

**Published:** 2017-05-23

**Authors:** Dennis Slate, Jordona D. Kirby, Daniel P. Morgan, Timothy P. Algeo, Charles V. Trimarchi, Kathleen M. Nelson, Robert J. Rudd, Adam R. Randall, Mark S. Carrara, Richard B. Chipman

**Affiliations:** 1 National Rabies Management Program, Wildlife Services, Animal and Plant Health Inspection Service, United States Department of Agriculture, 59 Chenell Drive, Suite 2, Concord, NH 03301, USA; Dennis.Slate@aphis.usda.gov (D.S.); jordona.d.kirby@aphis.usda.gov (J.D.K.); kathleen.m.nelson@aphis.usda.gov (K.M.N.); richard.b.chipman@aphis.usda.gov (R.B.C.); 2 New York Program, Wildlife Services, Animal and Plant Health Inspection Service, United States Department of Agriculture, 230 Timerman Hall, SUNY Potsdam, Potsdam, NY 13676, USA; dan.p.morgan@aphis.usda.gov; 3 Laboratory of Zoonotic Disease and Clinical Virology, Griffin Laboratory, Wadsworth Center, New York State Department of Health, 61 Mordella Road, Albany, NY 12205, USA; chucktrimarchi@yahoo.com; 4 Rabies Laboratory, Griffin Laboratory, Wadsworth Center, New York State Department of Health, 5668 State Farm Rd., Slingerlands, NY 12159, USA; robert.rudd@health.ny.gov; 5 New Jersey Program, Wildlife Services, Animal and Plant Health Inspection Service, United States Department of Agriculture, Pittstown, NJ 08867, USA; adam.r.randall@aphis.usda.gov; 6 Forest Service, United States Department of Agriculture, Cedar City, UT 84721, USA; mcarrara@fs.fed.us

**Keywords:** raccoon, skunk, rabies, enhanced rabies surveillance, road kill, oral rabies vaccination (ORV), meso-carnivores

## Abstract

Oral rabies vaccination (ORV) requires knowledge of the spatial-temporal distribution of rabies virus variants targeted for control. Rabies-exposure based public health surveillance alone may not provide a sound basis for ORV decisions. The value and cost of road kill surveys was evaluated for the late spring–early fall 2005–2007 as a part of enhanced rabies surveillance in northern New York, where raccoon rabies is enzootic and ORV has occurred since the late 1990s. Structured surveys were conducted to collect raccoons and other meso-carnivores for rabies testing at the New York State Rabies Laboratory. Of the 209 meso-carnivore heads collected and submitted for testing, 175 were testable by direct fluorescent antibody; none was rabid. Rabies was also not reported through public health surveillance in survey zones during 2005–2007. Overall, survey costs were $37,118 (2016 USD). Salaries and benefits accounted for 61% of costs, followed by fuel (22%), vehicle depreciation (14%), and sample shipping (3%). Mean daily distance driven was 303 ± 37 km and 381 ± 28 km for total road kills and raccoons, respectively. Costs/road kill collected and submitted was $176/all species and $224/raccoon. This study provides costs for planning road kill surveys and underscores the need to continually improve enhanced rabies surveillance approaches to support ORV decision making.

## 1. Introduction

Rabies surveillance is characterized as being active or passive [1]. Much of the rabies surveillance in the U.S. is based on exposure events brought to the attention of public health officials [2]. While effective in protecting the public from rabies, passive surveillance is inadequate to delineate the spatial-temporal distribution of rabies in meso-carnivore reservoirs such as the raccoon (*Procyon lotor*) in near real time [3].

Enhanced rabies surveillance is characterized as a form of surveillance with special emphasis on specific areas, species, and rabies management goals [4]. Since 2005, enhanced rabies surveillance has been extensively used in proximity to oral rabies vaccination (ORV) zones in the USA to increase sampling scope and intensity to delineate rabies distribution among wildlife rabies reservoir species for improved management.

Enhanced rabies surveillance serves as a complement to exposure-based public health surveillance, and collectively this information provides a more comprehensive spatial-temporal view of rabies for strategic ORV decisions to achieve rabies management goals in the U.S. Enhanced rabies surveillance targets a range of sample sources within or near (≤80 km) ORV zones, without a known human or domestic animal exposure history.

Sampling scope includes:

1. Animals exhibiting unusual behaviors suggestive of rabies brought to the attention of U.S. Department of Agriculture (USDA), Animal and Plant Health Inspection Service (APHIS), Wildlife Services (WS), or collected by program cooperators; these typically have the highest rabies prevalence among animals not involved in human or domestic animal exposure events;
2. Animals found dead (other than road kills);
3. Road kills from formal or opportunistic surveys;
4. Animals WS trapped and euthanized or shot in specified raccoon rabies risk areas, often where recent cases warrant sampling;
5. Animals captured by Nuisance Wildlife Control Operators (NWCOs) or others near ORV zones [4,5].

Road kill surveys have been conducted for a variety of wildlife management purposes, such as indexing population trends and abundance [6,7,8,9]; wildlife disease monitoring [10]; and characterization of population genomics [11]. We assessed the relative value of structured road kill surveys as a complement to public health surveillance to determine the presence or absence of raccoon rabies variant in raccoons and other meso-carnivore species into which spillover of this rabies virus variant occurs [12] (striped skunk (*Mephitis mephitis*), red fox (*Vulpes vulpes*), gray fox (*Urocyon cinereoargenteus*), coyote (*Canis latrans*), and fisher (*Martes pennanti*)) in northern New York. ORV had been conducted since 2001 (USDA, APHIS, Wildlife Services 2001) throughout much of the area where road kill surveys were conducted. These surveys were evaluated in the context of the cost of samples acquired and their contribution to rabies surveillance in the areas surveyed.

## 2. Materials and Methods

**Figure 1:**
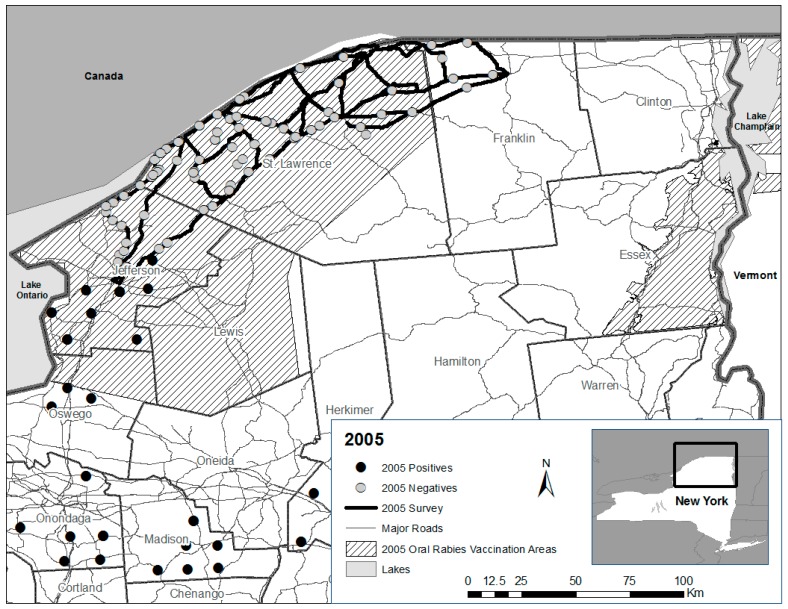
Rabies negative road kills collected during surveys (27 June–21 September) in Jefferson, St. Lawrence, and Franklin counties in 2005 relative to the oral rabies vaccination zone and public health positive cases.

**Figure 2:**
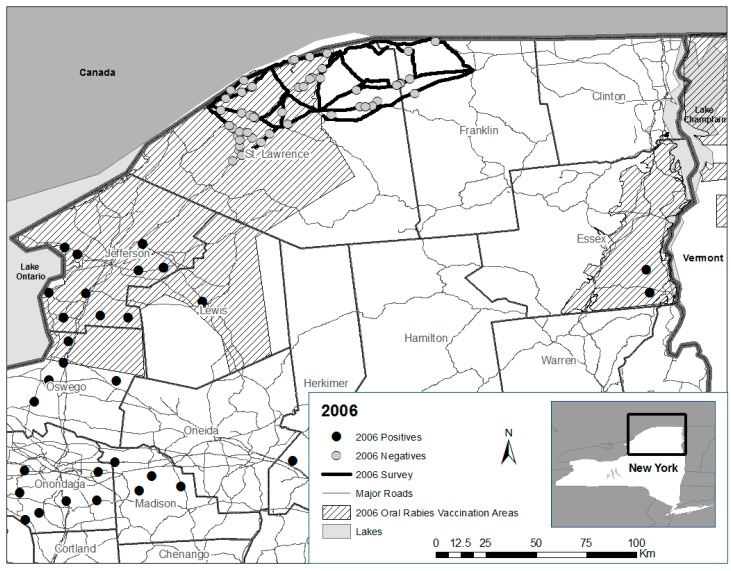
Rabies negative road kills collected during surveys (5 June–17 August) in St. Lawrence and Franklin counties in 2006 relative to the oral rabies vaccination zone and public health positive cases.

**Figure 3:**
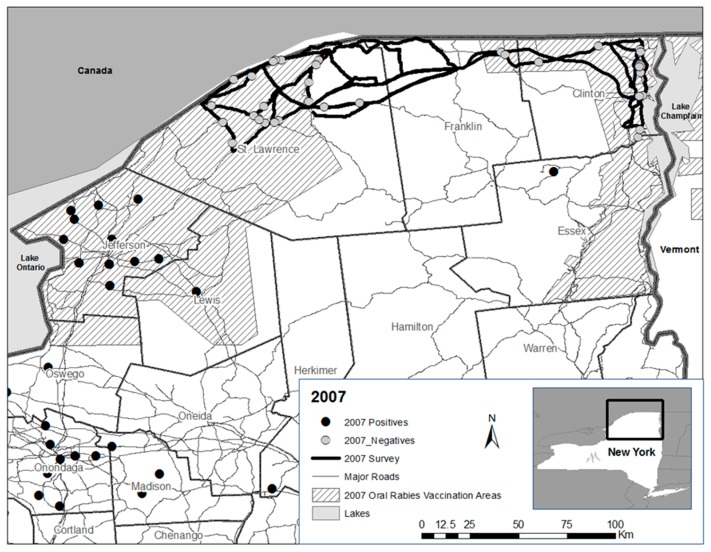
Rabies negative road kills collected during surveys (25 June–27 July) in St. Lawrence, Franklin, and Clinton counties in 2007 relative to the oral rabies vaccination zone and public health positive cases.

From 2005 to 2007, formal road kill surveys were initiated within or near ORV zones in northern New York (Figure 1, Figure 2 and Figure 3). Differences in routes among years were necessary in response to staffing availability. The same two personnel conducted nearly all the road kill surveys from 2005 to 2007. All personnel who participated in the road kill surveys received the recommended rabies pre-exposure vaccine series prior to conducting surveys.

Personnel were trained so that activities were uniformly conducted in a coordinated fashion, although there was not a strict protocol for speed driven by highway type or road-scanning breadth, as these may vary for safety, road shoulder width, and roadside mowing activities. Surveys were conducted in full-sized pickup trucks, which provided an improved road/road shoulder view over lower-framed vehicles.

Survey routes were driven from 6:00 am until approximately noon. State, County, and Municipal highway officials were contacted to inform them of the road kill survey times. Species subject to collection included: raccoon, skunk, foxes (red and gray), coyote, and fisher.

**Figure 4:**
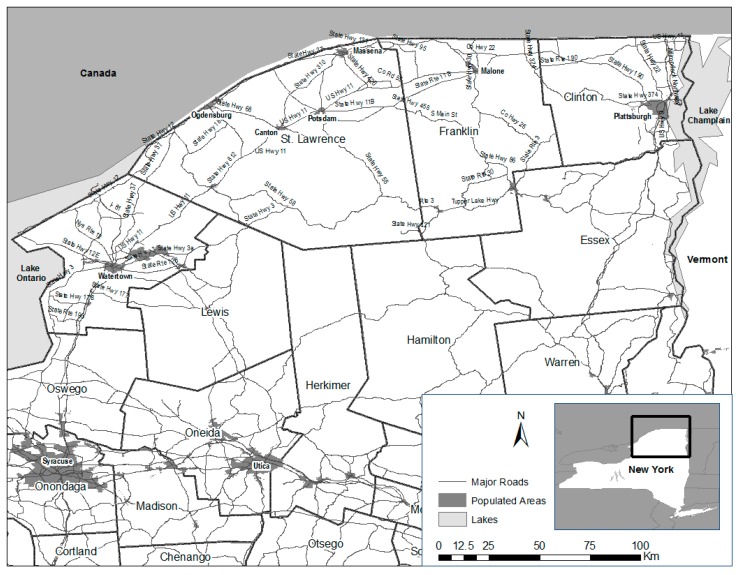
Reference map for counties, villages and cities, and routes that occurred within road kill survey areas, New York.

In 2005, specified routes were surveyed four days/week for 10 weeks from 27 June to 21 September (Figure 1). For staffing efficiency, the road kill survey area was divided into approximately equal east and west zones. The western zone was bordered on the west by U.S. Interstate 81 from Watertown (Jefferson County) to the St. Lawrence River, and State Route 812 between Ogdensburg and the intersection of State Route 11 near DeKalb on the east. The eastern zone included major roads (State Routes 37 and 11B) from this point East to Malone, which encompassed portions of St. Lawrence and Franklin Counties where ORV had not occurred (Figure 4). Both areas were further divided so that half of each area was sampled two days/week with minor exceptions, by two personnel (Tuesday and Thursday; and Wednesday and Friday).

In 2006, the portion of the road kill survey zone in Jefferson and southwestern St. Lawrence Counties was discontinued, reducing the zone that was sampled in 2005 by about half. The new 2006 zone was then divided approximately in half. However, unlike in 2005, each half was not further sub-divided and the entire zone was surveyed four days/week by two personnel with minor exceptions from 5 June to 17 August (Figure 2).

In 2007, the 2006 road kill survey zone was extended to include portions of eastern Clinton County, with State Routes 190 and 11 surveyed for road kills traveling to and from Franklin into Clinton County, and U.S. Interstate 87 and State Routes 9 and 22 making up much of the road matrix surveyed in the northern Lake Champlain Valley (Figure 3). Due to staffing demands, road kill surveys were conducted four days/week by two personnel during the period 25 June–27 July—a five, rather than a 10-week survey.

During sample collection, personnel wore appropriate personal protective equipment including nitrile gloves, hard hats, and safety vests for compliance with permits issued by the New York State Department of Transportation. When road kill carcasses were observed, personnel quickly and safely parked alongside the roadway to retrieve the carcass. Once personnel moved to a safe location, each carcass was inspected to determine if a viable sample could be collected and submitted for testing. For carcasses deemed viable, GPS coordinates were recorded for the road kill location, unique numbered ear tags were attached, and the whole head was removed at the base of the neck using heavy duty brush loppers. Samples were frozen until shipping.

Samples were prepared and packaged for shipment to the New York State Department of Health (NYSDOH) Rabies Laboratory at Wadsworth Center, New York on Monday of each week. Intact head samples were wrapped in newspaper and double-bagged (resealable plastic bags) separately with specimen labels containing the sample ear tag numbers. Multiple intact head specimens were included in each shipment in double-walled, insulated containers, in accordance with the UN-3373 Biological Specimen Category regulations. The following data were entered into the WS National Rabies Management Program (NRMP) database: date and GPS coordinates of sample collection; species; sex; relative age (juvenile or adult); and reproductive status.

Samples were categorized for data analysis as *detected, submitted*, and *tested*. Samples *detected* represented species of interest found on survey routes that often included animals deemed not suitable to be submitted for testing. *Submitted* samples included those that field personnel collected and determined to be candidates for rabies testing. NYSDOH Rabies Laboratory personnel made the final determination if submitted samples were suitable to be *tested* for rabies. The direct fluorescent antibody (dFA) test was used to determine the rabies status of road kills [13]. In 2005, road kills detected but considered unsuitable for rabies testing by WS staff were not recorded. All three categories were recorded in 2006 and 2007.

Direct or estimated costs for four major components of road kill surveys included staff salary and benefits [14], fuel costs [15], vehicle depreciation, and sample shipment costs adjusted to 2016 U.S. dollars.

Statistical Analysis System (SAS) 9.3 (SAS Institute, Cary, NC, USA) statistical software was used for analyses [16].

## 3. Results

Six meso-carnivore species were observed during road kill surveys in the St. Lawrence River Valley (Jefferson and St. Lawrence Counties), in northern Franklin County, and the northern Lake Champlain Valley (Clinton County) along the U.S.-Canada border from 2005 to 2007. Raccoons were most frequently detected (*n* = 320), followed by striped skunks (*n* = 136), and red foxes (*n* = 13) (Table 1). These were the only species that were submitted for rabies testing in all three years. Two gray foxes were detected and submitted during 2006 that were deemed not suitable for testing by the NYSDOH. A single fisher was submitted and tested negative in 2007, but was excluded from statistical analysis because of low sample size. None of the 140 raccoons, 26 skunks, 8 red foxes, and 1 fisher (*n* = 175) tested over the survey period was rabid by dFA.

In 2006 and 2007, 297 and 177 total road kills were detected, and 70 (24%) and 59 (33%) were deemed suitable to be submitted, with 58 (83%) and 37 (63%) tested, respectively. Raccoons accounted for most of the detected road kills in 2006 and 2007 at 196 (66%) and 124 (70%), respectively; 59 (30%) and 45 (36%) of detected raccoons were submitted for testing, with 50 (85%) and 28 (62%) of submitted raccoons tested, respectively. In 2005, road kills detected and deemed not suitable for testing were not recorded, but all 80 samples submitted were tested for rabies.

**Figure 5:**
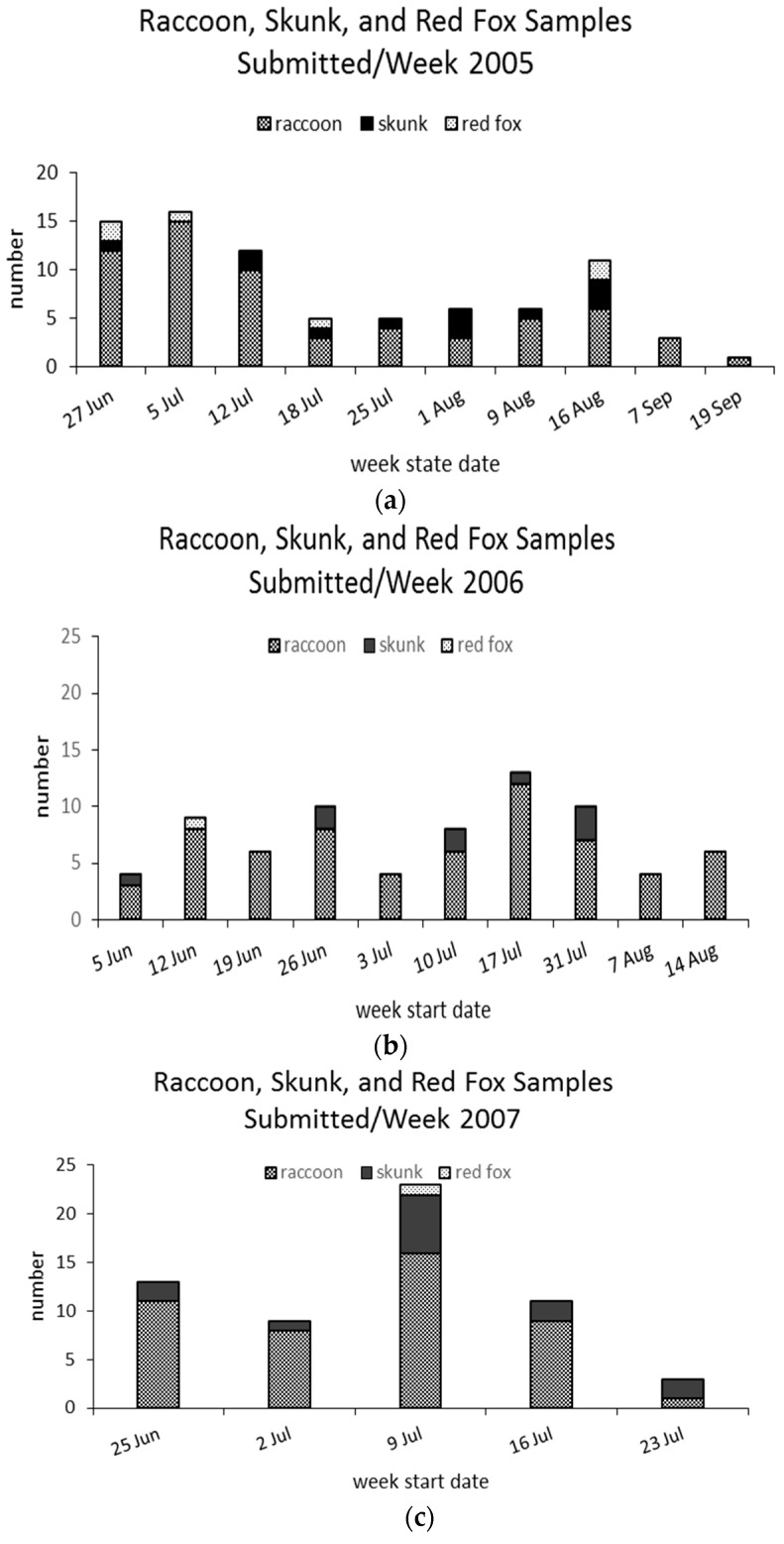
Number of raccoons, striped skunks, and red foxes submitted weekly for potential rabies diagnostics to the New York State Rabies Laboratory at Wadsworth Center, Albany, New York from road kill surveys in (**a**) 2005, (**b**) 2006 and (**c**) 2007.

The raccoon was the only species that occurred in all weekly samples from 2005 to 2007 (Figure 5a–c); the striped skunk occurred in an average of 73% of weekly samples during 2005–2007. Skunk representation among road kills was not different (*p* = 0.03) among the survey years relative to raccoons and averaged 19% of road kill raccoon submitted for testing annually.

**Figure 6:**
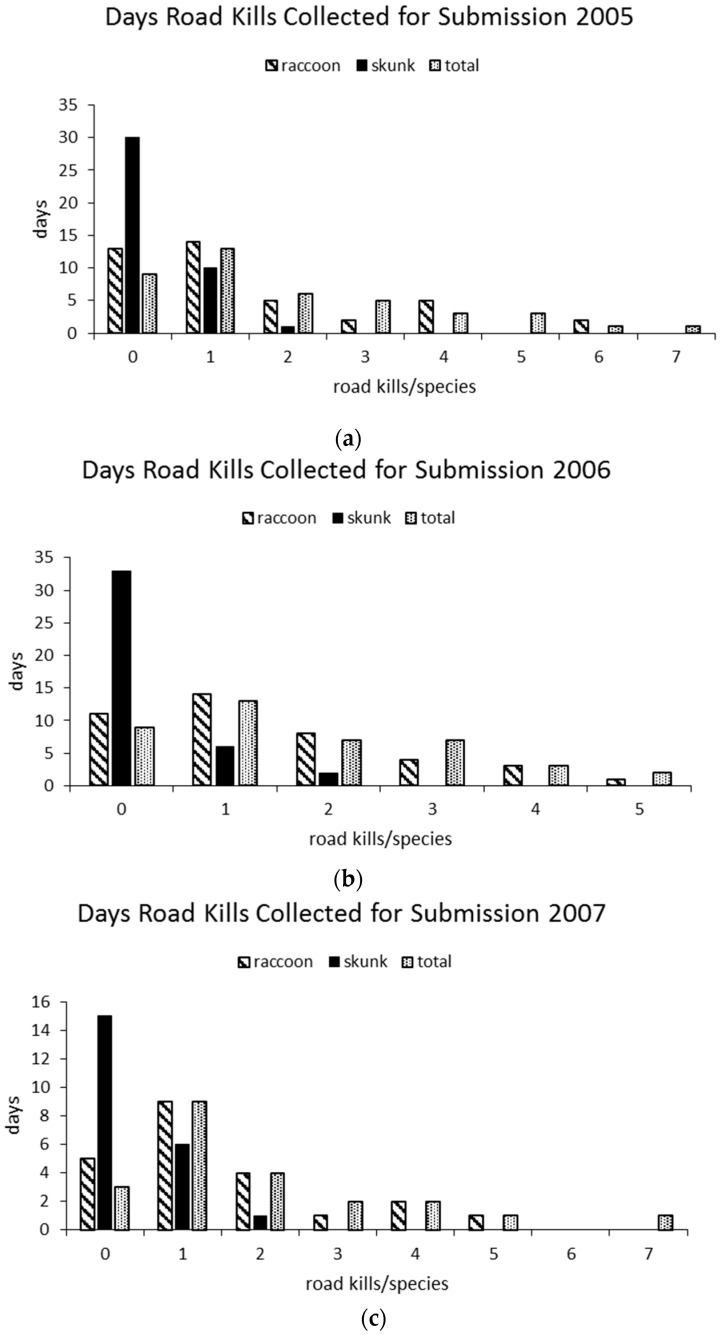
Frequency of days where raccoon, skunk, and total road kills collected for submission ranged from 0 to 7 (total may include red or gray fox, coyote, or fisher) in (**a**) 2005, (**b**) 2006 and (**c**) 2007.

On average, 19% of survey days resulted in collection of no road kills to be submitted for testing, ranging from 14% (*n* = 3/22) in 2007 to 22% (*n* = 9/41) in 2005 and 2006. No road-killed raccoons were collected for submission an average of 27% of survey days, ranging from 23% (*n* = 5/22) in 2007 to 32% (*n* = 13/41) in 2005. No road-killed skunks were collected for submission an average of 74% survey days, ranging from 68% (*n* = 15/22) in 2007 to 81% (*n* = 33/41) in 2006 (Figure 6a–c).

On average, 35% of survey days resulted in collection of one road kill that was submitted for testing, ranging from 32% in 2005 and 2006 to 41% in 2007. One road-killed raccoon was collected and submitted for testing an average of 36% of survey days, ranging from 34% (*n* = 14/41) in both 2005 and 2006 to 41% (*n* = 9/22) in 2007. One road-killed skunk was collected and submitted an average 22% of survey days, ranging from 15% (*n* = 6/41) in 2006 to 27% (*n* = 6/22) in 2007. Seven total road kills were collected for submission on single days in 2005 and 2007, which also included either red foxes or a fisher. The highest number of raccoons collected/day to be submitted for testing was six, which occurred on two days in 2005 (Figure 6a).

The mean daily km driven/raccoon collected for submission from 2005 to 2007 was 381 ± 28 (SD) for raccoons, 2099 ± 845 (SD) for skunks, and 303 ± 37 (SD) for total road kills. On the average, there was greater than a 5-fold increase in driving distance to collect a skunk to be submitted for rabies testing in comparison to a raccoon. A mean of 1.5 (range 0–6) raccoons was collected/day in all years; road-killed skunks collected/day averaged 0.4, 0.2, and 0.6 (range 0–2) in 2005, 2006, and 2007, respectively.

Simple linear regression revealed a significant (*p* < 0.02) positive relationship between weekly km driven and road kills collected in 2005 only. Weekly km driven accounted for about half of the variation (r^2^ = 0.52) in the dependent response weekly road kills that were collected and submitted for testing.

**Figure 7:**
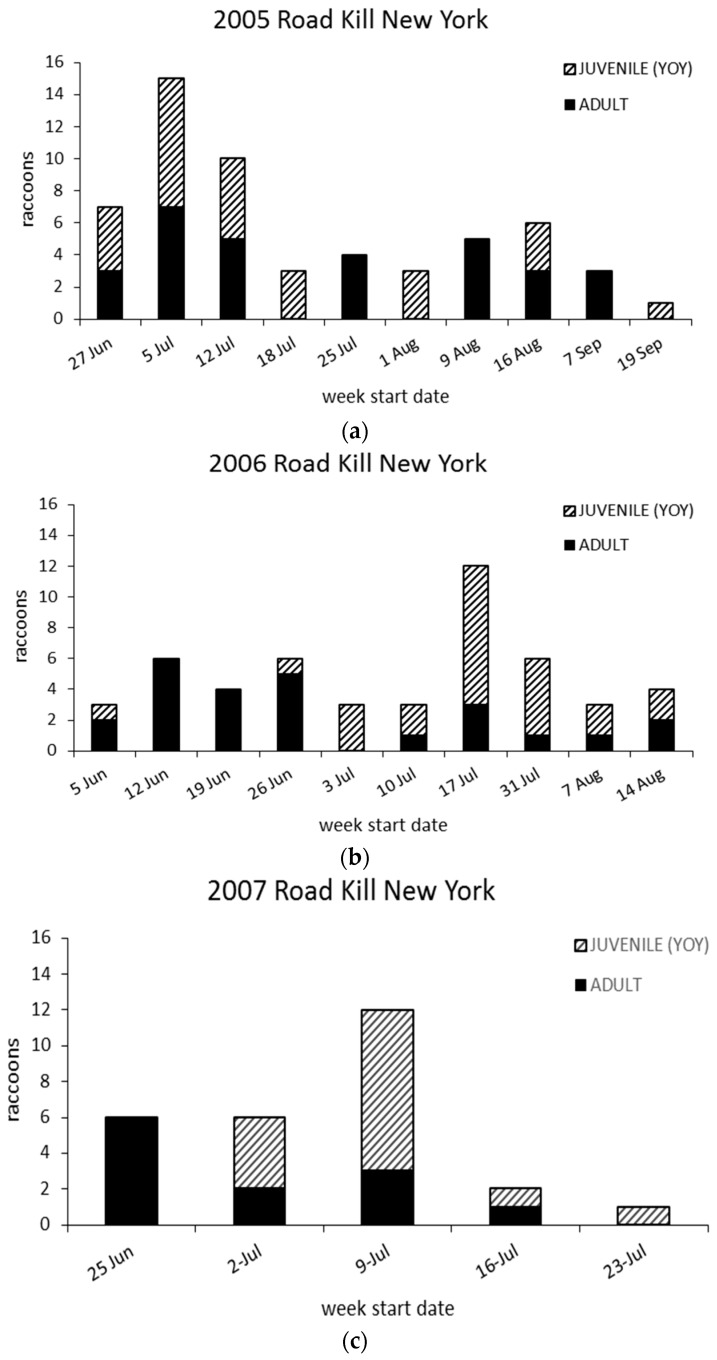
Adult and juvenile (young of the year) raccoons collected deemed suitable for submission for rabies testing (**a**) 2005, (**b**) 2006 and (**c**) 2007.

Weekly surveys resulted in increasing samples of juvenile raccoons during July, when young often begin traveling more extensively in family groups at north temperate latitudes [17] (Figure 7a–c). There was no difference (*p* = 0.82) in sex and relative age among road-killed raccoons within and across survey years. While there were more adult females and juveniles collected and tested in all survey years, there was not a significant association (*p* = 0.48) when juveniles were pooled with adult females in comparison to adult males.

Road kill surveys required a commitment of two trained wildlife staff for 25 weeks over the survey period. Cumulative mean weekly driving distances ranged from 2372 km in 2005 to 3169 km in 2007, requiring a mean range of 35 to 50 h driving and sample preparation time. Annual variation in survey routes and adaptive changes resulted in more kilometers (km) driven over five days in 2007 than 10 days in 2005. The survey area was larger in 2005 and 2007 than 2006, but the 2005 survey area was split in half and each half only driven two days/week.

Costs associated with road kill surveys from 2005 to 2007 in 2016 USD in decreasing order of importance were: salaries and benefits ($22,778; 61%), fuel ($8142; 22%), vehicle depreciation ($5274; 14%), and sample shipping ($924; 3%) (Table 2). The cost/road kill collected to be submitted for testing was: $176/all species, $224/raccoon, and $1160/skunk. While we did not estimate rabies diagnostic and associated laboratory costs for testing road kills, the NYSDOH Rabies Laboratory approximates the cost for rabies testing/specimen at $150.00 in 2016. This cost does not include equipment depreciation, training, safety measures, administrative burden, database management, and other related costs that would be included in a comprehensive laboratory cost analysis.

## 4. Discussion

The stated goals of surveillance include: (1) detection of the potential emergence of a disease as early as possible; (2) characterization of the spatial-temporal distribution of a disease once it is detected; (3) determination of factors that contributed to its emergence and distribution; and (4) determining whether the disease has disappeared or been controlled or eliminated through intervention [1].

Rabies surveillance in the U.S. has been and continues to be predominantly based on human and domestic animal exposures as a priority to ensure that public health is protected against this invariably fatal disease once clinical signs manifest. With the integration of ORV into rabies control in wild meso-carnivores, there is a management need for a more comprehensive near real-time view of the spatial-temporal distribution of specific rabies virus variants. Road kills as well as other sources of samples (e.g., strange acting animals not involved in human or domestic animal exposures, animals found dead in addition to road kills) not traditionally tested for rabies provide greater spatial-temporal surveillance scope and intensity than public health exposure-based samples alone for improved ORV decision making [4]. None of the 175 wild meso-carnivores tested from road kill surveys during 2005–2007 was rabid by dFA. During the same period, no meso-carnivores were tested or reported rabid by the NYSDOH Rabies Laboratory within the road kill survey zone; a single rabid raccoon was reported by NYSDOH near the edge of the road kill zone in 2005, northeast of Watertown, NY, USA (Figure 1). That no rabid terrestrial mammals were reported during the road kill survey period may have reflected suppression or elimination of raccoon rabies at the multiple county level, which may not be unexpected given that ORV intervention with RABORAL V-RG^®^ baits (Merial Ltd., Athens, GA, USA) had been annually in place in portions of northern Jefferson and southern Clinton counties since 1995; portions of St. Lawrence and Franklin Counties were added in 1997 and 1998, respectively, along with a portion of northern Clinton County in 2007.

**Figure 8:**
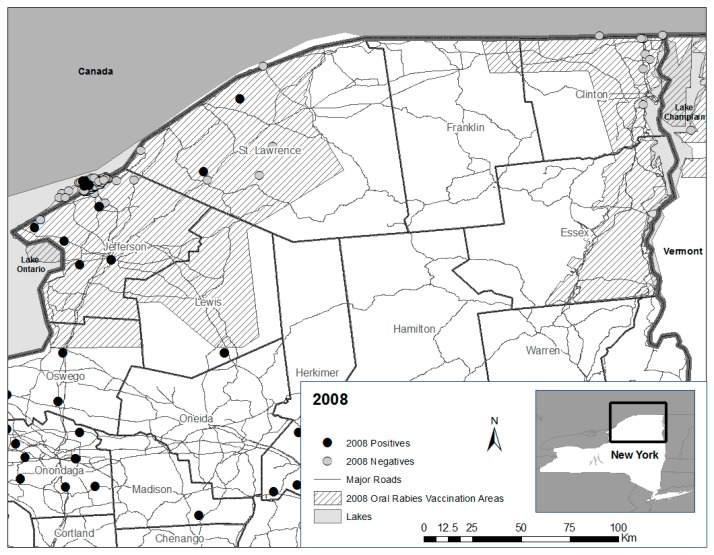
Distribution of raccoon rabies cases in 2008 in northern New York within and outside of long standing oral rabies vaccination (ORV) zones.

However, reemergence of raccoon rabies within northernmost Jefferson and portions of St. Lawrence counties in 2008 (Figure 8), where ORV continued annually but where road kill surveillance ceased as a complement to public health surveillance points to two potential issues, independently or in combination.

(1) It is not known if raccoon rabies had been transiently eliminated as suggested by enhanced surveillance through road kills in combination with public health surveillance, or if enhanced surveillance in combination with public health surveillance was insufficient to detect raccoon rabies presence that may have been suppressed by ORV or other factors; (2) Also, it is not known if vaccine-baits or implemented strategies (or their interaction), given generally low rabies virus neutralizing antibody seroprevalence observed in raccoon populations post-ORV [5], were adequate to contribute to lasting raccoon rabies suppression or perhaps elimination at this county scale given the perpetuation of rabies cases within the contiguous ORV zone that extended south beyond road kill survey areas in Jefferson County (Figure 1, Figure 2 and Figure 3 and Figure 5).

The ultimate goal of the NRMP is elimination of specific terrestrial rabies variants, including raccoon rabies [18]. The detection of rabies within this specific survey area beginning in 2008 does not alter this goal or negate the value of road kills as a form of enhanced rabies surveillance or ORV as a means of controlling and eliminating specific rabies variants. Rather, this outcome underscores the need to continually seek improvements in enhanced rabies surveillance approaches by emphasizing detection of strange behaving raccoons and other meso-carnivores not involved in human or domestic animal exposures to facilitate improved ORV decisions to achieve national wildlife rabies management goals of control and elimination [19].

Road kill collection was not inexpensive at $176/road kill and $224/road-killed raccoon over the three-year survey period. However, we are not aware of systematic economic analysis for rabies surveillance in the U.S. to formally compare the relative cost of road kill samples to other rabies surveillance sample categories (e.g., suspect strange behaving raccoons).

Opportunistic road kill sampling, in lieu of more structured road kill surveys, represents an alternate if areas of interest can be adequately sampled through routine travel to and from the office and field work sites by experienced personnel over regular routes. Such sampling may better integrate into surveillance budgets without compromising area coverage and effort. Also, eliciting cooperation from personnel within transportation departments who may have the responsibility of removing road kills should be explored [20], so long as protective measures are taken to ensure against rabies exposure from carcass collection. In some instances, state wildlife conservation officers or game wardens may be an option for road kill sampling, given the extensive amount of travel required to accomplish their job duties.

This study served in part as a basis for future enhanced rabies surveillance planning. While it accomplished that goal, study limitations of note included the lack of consistency across years in road kill survey routes and effort due to changing staffing needs for rabies control in New York and the eastern U.S. Also, we did not attempt to predict road kill frequency based on estimated raccoon population density indices, traffic volume, or road type (i.e., primary, secondary, tertiary). The roads driven in these surveys were largely state routes and some county connecting roads, with small segments of Interstates 81 and 87 in 2005 and 2007, respectively (Figure 5a,c). For context, estimated daily traffic volume (number of vehicles) for select roads within the St. Lawrence County portion of road kill surveys in 2006 was: 4900 for the end of Route 812 at Route 11; 620 at the end of Route 58 at Route 37; 2530 from Route 37 west of the City of Ogdensburg; and, 11,770 at the start of Route 11 in Canton [21]. Raccoon population density indices were available only from 1998 to 2000 from three sites in St. Lawrence County that ranged from 2 to 8 animals/km^2^ (USDA unpublished data). Given these examples of variation in estimated traffic volume and raccoon densities, their potential value in determining expected sample size/effort for future road kill surveys should be explored. We also did not record animals detected and discarded that were deemed unsuitable to be submitted for rabies testing in 2005. That additional year would have better characterized variation in the proportion of road-killed animals submitted for testing relative to the total detected. The differential between road kills detected and collected serves as a measure of efficiency and is particularly informative for road kills detected on day one that may be unsuitable because they were killed during the weekend and were cleared from the highway by WS on Monday because of extended exposure to road traffic, heat, scavenging, or other factors. At more southern latitudes where road kills quickly degrade in high temperatures, the ratio of animals submitted:detected may be useful to delineate times when road kill surveys may not be a wise commitment of resources. Finally, there is a strong suspicion that the abnormal behaviors commonly associated with clinically rabid raccoons and other animals increase their likelihood of being killed on highways above that observed for healthy subjects. However, to test this hypothesis would require additional well-designed field studies.

In 2005, 2006, and 2007, during a raccoon rabies epizootic in northeast Ohio, road kills ranked third among five enhanced rabies surveillance categories for detection of rabies (2/399, 0/159, 1/481). Strange behaving raccoons not involved in human or domestic animal exposures had the highest probability of rabies detection (17/578, 5/263, 15/191), followed by animals found dead not as road kills (2/142, 1/97, 3/106). NWCO-captured, apparently healthy animals ranked last at (0/1680, 0/17, 0/12) for the three years (no rabies positives in 2005 and direct rapid immunohistochemistry test (dRIT) WS laboratory burden led to reduced testing of NWCO derived samples in 2006 and 2007), with WS trapped and tested animals from within or near the epizootic ranking number four (1/738, 0/296, 0/90) [22]. While all of these enhanced rabies surveillance categories may be valuable during rabies emergence requiring ORV intervention, road-killed raccoons and other meso-carnivores represents a dependable source of samples that appears to add value to enhanced rabies surveillance in combination with strange acting animals and animals found dead. Collectively, enhanced, and public health rabies surveillance provides a more complete spatial-temporal view of rabies distribution on which to make the most informed ORV decisions for controlling rabies in wildlife.

## Figures and Tables

**Table 1:** Summary of road kill samples detected, submitted, and tested for rabies from road kill surveys conducted in northern New York during late spring-early fall 2005–2007.

Year ^1,2^	2005 ^3^			2006			2007		
Species	Detect	Submit	Test	Detect	Submit	Test	Detect	Submit	Test
Raccoon		62	62	196	59	50	124	45	28
Striped Skunk		12	12	90	8	7	46	12	7
Red Fox		6	6	8	1	1	5	1	1
Gray Fox		0	0	2	2	0	0	0	0
Coyote		0	0	1	0	0	1	0	0
Fisher		0	0	0	0	0	1	1	1
Total		80	80	297	70	58	177	59	37

^1^ Some of the road kill areas varied among years. ^2^ Survey dates were: 27 June 2005–21 September 2005; 5 June 2006–17 August 2006; 25 June 2007–27 July 2007. ^3^ Road kill samples detected that were unsuitable to be submitted to the rabies laboratory were not recorded in 2005 only; all samples submitted in 2005 were tested for rabies by direct fluorescent antibody (dFA).

**Table 2:** Estimated annual cost of formal road kill surveys in Northern New York State in 2005–2007 (all values are shown in 2016 USD).

Year	Salary and Benefits ^1^	Fuel ^2^	Vehicle Depreciation ^3^	Sample Shipping ^4^	Totals
2005	8306	3104	1812	389	13,611
2006	8322	3043	1755	351	13,471
2007	6150	1995	1707	184	10,036
Totals	22,778	8142	5274	924	37,118

^1^ Salary hourly rates for 2016 for the employee grades (GS 5_1_, 6_1_, 6_3_, 7_1_, and 7_2_) for two personnel who conducted almost all of the road kill surveys and prepared samples for overnight pick-up and shipping, plus a mean benefit rate of 0.35 [14]. ^2^ Includes five month late spring-early fall mean for the price of a US gallon of gasoline plus taxes based on 2006 ($302.40) and 2007 ($304.80) pump rates at an estimated 7.2 km/L (17 mi/gal). [15]. The 2006 rate was also applied for 2005, which was not readily available. ^3^ A simple linear annual rate based on Blue Book value for the vehicle with 150,000 mi “life expectancy” in 2016. ^4^ Based on $14.50/shipment from Potsdam, NY to the NYSDOH Rabies Laboratory, Wadsworth Center, Slingerlands, NY, where total heads submitted/2.5 shipments ≥ 2 then an estimated 2.5 shipments occurred/week; otherwise 1 or no shipments occurred/week.

## Abbreviations

APHIS	Animal and Plant Health Inspection Service
dFA	direct fluorescent antibody
NRMP	National Rabies Management Program
NYSDOH	New York State Department of Health
NWCO	Nuisance Wildlife Control Operator
ORV	oral rabies vaccination
USDA	U.S. Department of Agriculture
WS	Wildlife Services
